# Seasonal variation in thermal tolerance and hypoxia tolerance of a threatened minnow and a non-imperilled congener: a cautionary tale for surrogate species in conservation

**DOI:** 10.1093/conphys/coae071

**Published:** 2024-10-16

**Authors:** Jessica E Reemeyer, Dominique Rumball, Nicholas E Mandrak, Lauren J Chapman

**Affiliations:** Department of Biology, McGill University, 1205 Dr Penfield Avenue, Montreal, Quebec, Canada; Department of Biological Sciences, University of Toronto Scarborough, 1265 Military Trail, Toronto, Ontario, Canada; Department of Physical and Environmental Sciences, University of Toronto Scarborough, 1265 Military Trail, Toronto, Ontario, Canada; Department of Biological Sciences, University of Toronto Scarborough, 1265 Military Trail, Toronto, Ontario, Canada; Department of Physical and Environmental Sciences, University of Toronto Scarborough, 1265 Military Trail, Toronto, Ontario, Canada; Department of Biology, McGill University, 1205 Dr Penfield Avenue, Montreal, Quebec, Canada

**Keywords:** Pugnose shiner, respirometry, species at risk, surrogate species

## Abstract

Freshwater organisms face multiple threats to their ecosystems, including warming associated with climate change and low dissolved oxygen (environmental hypoxia), which are both increasing in frequency and extent in freshwater systems. Understanding tolerance thresholds for these environmental stressors as well as the plasticity of responses is the key for informing the conservation of imperilled species. Direct measurement of imperilled species can be difficult, and the use of surrogate (non-imperilled but closely related) species has been proposed as a remedy, but the degree to which surrogate data are representative of the imperilled species has not been widely validated. In this study, we measured physiological performance of two species: one federally listed as Threatened in Canada (Pugnose Shiner, *Miniellus anogenus*) and a non-imperilled congener (Blackchin Shiner, *Miniellus heterodon*). Hypoxia tolerance (critical oxygen tension and loss of equilibrium) and upper thermal tolerance (CT_max_) were measured streamside over a period of 5 months. We found that the Threatened Pugnose Shiner had lower tolerance to both elevated temperature and hypoxia than the non-imperilled Blackchin Shiner. The species also differed in their responses to environmental dissolved oxygen (DO). CT_max_ of Pugnose Shiner had a positive relationship with DO such that CT_max_ was lowered when environmental DO was low, whereas there was no effect of DO on CT_max_ of Blackchin Shiner. Blackchin Shiner also showed plasticity of hypoxia tolerance in response to changes in environmental DO, while Pugnose Shiner showed little plasticity. We conclude that Pugnose Shiner may be more sensitive to heat waves and hypoxia associated with climate change. We also assert that researchers should be cautious when using surrogate species to inform tolerance limits of imperilled species and highlight the value of measuring imperilled species directly when possible.

## Abbreviations

A, akaike information criterion
a.s., air saturation
CF, condition factor
CI, confidence interval
CT_max_, critical thermal maximum
DO, dissolved oxygen
LOE, loss of equilibrium
MO_2_, oxygen consumption rate
OAC, Old Ausable Channel
OCLTT, oxygen and capacity-limited thermal tolerance
*P*_crit_, critical oxygen tension
RMR, routine metabolic rate
s.e., standard error
SL, standard length
SMR, standard metabolic rate
*T*_ag_, agitation temperature

## Introduction

Fresh waters support nearly half of fish species globally while covering only a small fraction of the Earth’s surface (Strayer and Dudgeon, 2010). Unfortunately, freshwater systems face various anthropogenic threats associated with land development, pollution, altered flow, invasive species and changes in water quality (Dudgeon *et al.*, 2006; Strayer and Dudgeon, 2010; Vörösmarty *et al.*, 2010; Arthington *et al.*, 2016; Hermoso, 2017). As a result, it is estimated that 30% of freshwater fish species are at risk of extinction globally (WWF, 2021). Understanding drivers underlying the imperilment in these fishes is essential in developing conservation efforts.

Studying imperilled species directly can be challenging due to a variety of factors. At-risk species often have small, disconnected populations, individuals within these populations may be rare in the environment, and knowledge of how to sample and maintain individuals in a laboratory setting may be lacking. Moreover, development of this knowledge is resource intensive both financially and human-resource timewise. Compounding this, conservation budgets are often limited and require triaging of resource allocation (Bottrill *et al.*, 2009). The use of surrogate species, wherein a secondary species is studied/used in place of the target imperilled species, has been proposed to circumvent these issues and maximize the use of conservation dollars (Caro and O’Doherty, 1999; Wiens *et al.*, 2008; Caro and Girling, 2010). The assumption is that studying the effects of environmental stressors on a nontarget species, closely related to the target imperilled species, will be representative of effects on the target imperilled species (Caro *et al.*, 2005; Wenger, 2008). This method has not been widely validated and has been identified as a knowledge gap in the conservation of multiple freshwater fishes in Canada (Castañeda *et al.*, 2021).

Although there are many potential drivers of imperilment in freshwater fishes in Canada, climate warming has the potential to have profound effects on ectothermic organisms like fishes and is likely to interact with other co-occurring stressors such as hypoxia (McBryan *et al.*, 2013; Earhart *et al.*, 2022). In ectotherms, temperature determines the rate of physiological processes and performance (Huey and Stevenson, 1979). Fishes have a window of temperatures within which performance is optimized and beyond which performance declines (Huey *et al.*, 2012; Schulte, 2015). In northern latitudes, summer heat waves can lead to prolonged stressful temperatures beyond the optimal range of performance, which may affect species persistence (Huey *et al.*, 2012). Climate warming is expected to increase the frequency and duration of extreme heat events and increase the average and variability in temperature (Meehl and Tebaldi, 2004). Low dissolved oxygen (DO) (hypoxia) can also have a marked effect on fish performance, as oxygen is required for aerobic metabolism. Hypoxia is a pervasive stressor that is becoming more common in aquatic systems (Jenny *et al.*, 2016) and is expected to interact with warming waters in both cause and effect; higher temperatures increase the rate of eutrophication, while fish energy expenditure is increased by warming and limited by hypoxia. Understanding the interactive effects of these environmental stressors has been of interest in recent years (McBryan *et al.*, 2013; Earhart *et al.*, 2022) and is essential in predicting the effects of climate change in aquatic systems.

This present study aimed to assess the potential impact of elevated temperature and hypoxia on the physiological tolerance of two closely related species: Pugnose Shiner (*Miniellus anogenus*), which is listed as Threatened under Canada’s Species at Risk Act (Species at Risk Act S.C. 2002; COSEWIC, 2013), and the non-imperilled Blackchin Shiner (*Miniellus heterodon*). By comparing these species, we also aimed to validate the surrogate-species concept. We hypothesized that if Blackchin Shiner is an appropriate surrogate for Pugnose Shiner, their tolerances would be similar as well as the plasticity in these traits in response to changes in environmental temperature and oxygen. Over a 5-month period, fishes were sampled from the same population and habitat and measured for tolerance to elevated temperatures and hypoxia in the field. All measurements had nonlethal endpoints and allowed for fishes to be released after measurement.

## Materials and Methods

### Study species

Pugnose Shiner and Blackchin Shiner are both small-bodied minnows native to the upper Mississippi and Great Lakes basins in North America (Page and Burr, 2011). Pugnose Shiner was first listed as Endangered in 2003 and reclassified to Threatened in 2013 (COSEWIC, 2013), while Blackchin Shiner has no imperilled status. They are morphologically very similar. The main distinguishing feature is their mouth morphology; Pugnose Shiner has a more narrow and upturned mouth than Blackchin Shiner (Holm *et al.*, 2022). They are also ecologically very similar, often being positively associated during surveys (Carlson, 1997; Haynes *et al.*, 2019; Potts *et al.*, 2021a) and are, therefore, an ideal pair for testing the surrogate-species concept.

### Field site

The Old Ausable Channel (OAC) is a14-km long waterbody stretching from Grand Bend to Port Franks, Ontario along the south shore of Lake Huron, with most of the channel existing within the Pinery Provincial Park. The OAC was originally part of the Ausable River but was cut-off from it in the 1800s. Its disconnection from the Ausable River greatly reduced flow, and the OAC is now mostly groundwater and rainwater fed (Maun and Schincariol, 2010). This process also conferred protection from agricultural run-off in the area. The OAC is home to several at-risk fish species and is identified as critical habitat for the Threatened Pugnose Shiner and Endangered Lake Chubsucker (*Erimyzon sucetta*) (COSEWIC, 2013, 2021). Two fish species of Special Concern, Grass Pickerel (*Esox americanus*) and Northern Sunfish (*Lepomis peltastes*), are also present in the channel (COSEWIC, 2005, 2016).

**Figure 1:**
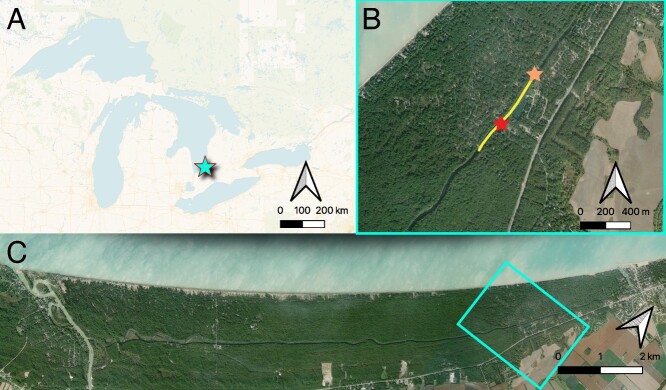
Location of study conducted on Blackchin Shiner and Pugnose Shiner. (A) shows the field location (cyan star) in relation to the surrounding Great Lake basin. (B) shows the area where fish collection occurred (yellow shaded area), the location of the research trailer where experiments took place (red star), and the surface temperature logger (orange star). (C) shows the location of panel B (cyan box) in relation to the rest of the Old Ausable Channel.

This study took place May–September 2021 near Grand Bend, Ontario, Canada. Our field site was located at the Huron Woods Community Centre (43.290429°, −81.779460°), just outside the Pinery Provincial Park boundary (Fig. 1). Captured fishes were held, and all physiological tolerance trials (detailed below) were performed, in a 4.9 m long portable research trailer parked channel side.

### Fish collection

Fishes were sampled in the channel using a modified boat-seining technique. Motorized vehicles are not permitted on the OAC, so a Jon boat with oars was used. A 30-m seine was deployed in a circle formation from the stern of a Jon boat. The net was then brought into the boat as one person pulled in the net while a second person constantly rowed to keep the boat stationary. All captured fishes were placed into an aerated bin. Fishes were identified to species by viewing them in a clear acrylic fish viewer, with target species being retained and bycatch being released immediately at the site of capture. Target fishes were brought back to the research trailer and held in an aerated cooler for up to 28 hours (until use in experiments detailed below).

All work was approved by the relevant institutional and governmental bodies (McGill University animal care protocol no. 7951; SARA permit no. PCAA-00020; OMNRF License to Collect Fish for Scientific Purposes No. 1100580).

### Environmental variables

Surface water temperature and DO were recorded throughout the field season. For water temperature, data were taken from a surface temperature logger placed by Ausable-Bayfield Conservation Authority less than 1 km upstream of our field site (Fig. 1). The logger recorded surface water temperature at 15-minute intervals. The mean, minimum, and maximum were then calculated for each day (24-hour period) of the field season. For DO, a handheld Polaris probe was used, and measurements were taken beside the field site from a dock once to twice daily in between physiological trial measurements.

### Thermal tolerance

Thermal tolerance was assessed as critical thermal maximum (CT_max_) (Becker and Genoway, 1979), following standard protocols. Fish were weighed, measured in a fish viewer, and placed into one of three baskets (15 cm length, 13 cm width, 12.5 cm height) within a larger tank (61 cm length, 30.5 cm width, 20.3 cm height) filled with water from the OAC and equipped with a recirculating heater (Julabo Corio CD, www.julabo.com) and an air bubbler. On days where DO in the OAC was lower than 80% a.s., the water was aerated and DO increased to 80% a.s. before fish were added to their basket. Fish were allowed to habituate to the tank for 1 h, and the trial was started wherein water temperature was raised at a rate of 0.3°C per min. The temperature at which the fish lost equilibrium for more than 3 consecutive seconds was recorded as CT_max_, and the fish was immediately removed from the set up and put in a recovery bath for at least 30 min before being released back into the channel near the site of collection. Individual fish behaviour was also monitored during the trial, and the first temperature at which the fish showed avoidance behaviour (darting quickly, seeking escape) was recorded as the fish’s agitation temperature (*T*_ag_) (McDonnell and Chapman, 2015; Wells *et al.*, 2016). Thermal safety margin was calculated as the difference between CT_max_ and the maximum environmental temperature of the day preceding CT_max_ measurement.

### Hypoxia tolerance

Hypoxia tolerance was measured using a widely used metabolic rate index (critical oxygen tension, *P*_crit_) (Rogers *et al.*, 2016). Whole organism oxygen consumption (MO_2_) was measured using intermittent flow respirometry (Svendsen *et al.*, 2016) using AutoResp software (www.Loligosystems.com). The system consisted of a Plexiglass aquaria (40.6 cm length, 40.6 cm width, 20.3 cm height) with four glass chambers (internal diameter 2.8 cm and 8 cm length) equipped with optical DO sensors, each connected to two submersible pumps forming two independent tubing loops: the ‘recirculating loop’, which circulates water within the chamber; and, the ‘flush’ loop that flushed water from the surrounding reservoir through the chamber (www.Loligosystems.com). Temperature was automatically maintained by the software by controlling a pump that flowed water from the reservoir through a coil submerged in ice.

The morning of a trial (~09:00), the respirometry set up was filled with water from the channel. Each fish was subsequently weighed, measured in a fish viewer and put into a respirometry chamber (~10:00) for at least 5 h during which their metabolic rate was measured periodically with a 90 s flush period, 30 s wait period and a measurement period that varied between 120 and 420 s depending on the mass and MO_2_ of the fish. The measurement period was adjusted according to the fish to maximize R^2^ of MO_2_ slope estimates while maintaining DO of > 80% air saturation (a.s.) in the chambers. After 5 hours, the chamber was sealed for a prolonged period wherein the DO in the chamber dropped until the fish lost equilibrium (the DO level at which was recorded as the fish’s loss of equilibrium (LOE)). The fish then recovered in fully aerated water for 30 minutes before being returned to the channel near the site of collection.

Due to logistical limitations, fish could not be measured overnight and, thus, it was not possible to measure standard metabolic rate (SMR), which has been suggested to be ideal for standardizing *P*_crit_ measurement (Reemeyer and Rees, 2019). Instead, routine metabolic rate (RMR) was used, which includes some spontaneous movement of the fish (Chabot *et al.*, 2016). RMR was calculated as the mean of the lowest 10% of MO_2_ measurements made from the time the fish was put into the chamber until the chamber was sealed for *P*_crit_ measurement. To measure *P*_crit_, the chamber was sealed for a prolonged period (31–148 min, mean: 73 min), wherein MO_2_ was sampled every 120–240 s. This *P*_crit_ measurement phase was ended when the fish lost equilibrium for at least 3 s, and the level of oxygen at which this happened was recorded as LOE. The chamber was then flushed, and the fish was allowed to recover for at least 5 minutes before removal from the chamber and placement in a bucket of aerated water, where the fish recovered for at least 25 minutes before being released back to the site of collection.

To control for background respiration of microbes within the chambers, the respirometry set up was sanitized between trials with dilute bleach. Measurement of background respiration occurred before and after measurement of fish with settings of 60 s flush period, 10 s wait period and 1200 s measurement period. For all trials, background respiration was 0 before and after fish measurement, so MO_2_ values were not background-corrected. A checklist following recent guidelines for reporting metabolic rate data is provided in Supplementary Table S1 (Killen *et al.*, 2021).

**Table 1:** Biometric data (mean ± s.e.) for Blackchin Shiner and Pugnose Shiner used in critical thermal maximum (CT_max_) measurements as well as environmental temperature the day before CT_max_ measurement (used to calculate Thermal Safety Margin)

**Species**	**Month**	**N**	**Mass (g)**	**SL (cm)**	**CF (g/cm** ^**3**^ **)**	**CT**_**max**_ **(°C)**	**Max Temp (°C)**
Blackchin Shiner	May	14	1.28 ± 0.15	4.3 ± 0.2	1.50 ± 0.05	33.9 ± 0.3	20.09 ± 0.38
	Jun	36	1.57 ± 0.09	4.4 ± 0.1	1.76 ± 0.03	35.3 ± 0.2	22.71 ± 0.35
	Jul	33	1.30 ± 0.09	4.3 ± 0.1	1.58 ± 0.02	35.1 ± 0.3	23.62 ± 0.11
	Aug	34	1.40 ± 0.09	4.3 ± 0.1	1.66 ± 0.03	35.7 ± 0.2	23.09 ± 0.14
	Sept	14	1.04 ± 0.13	4.0 ± 0.2	1.51 ± 0.04	34.0 ± 0.2	21.16 ± 0.17
Pugnose Shiner	May	15	0.63 ± 0.04	3.6 ± 0.1	1.41 ± 0.72	33.3 ± 0.2	19.82 ± 0.35
	Jun	26	1.08 ± 0.06	4.0 ± 0.1	1.67 ± 0.04	34.9 ± 0.2	22.25 ± 0.46
	Jul	6	1.03 ± 0.14	4.0 ± 0.2	1.51 ± 0.06	34.9 ± 0.8	23.69 ± 0.26
	Aug	16	0.96 ± 0.05	3.9 ± 0.1	1.59 ± 0.03	34.8 ± 0.4	23.73 ± 0.25
	Sept	18	0.56 ± 0.10	3.2 ± 0.2	1.50 ± 0.03	33.7 ± 0.4	21.05 ± 0.21

Fulton’s condition factor (CF) was calculated as mass divided by standard length (SL) cubed.

### Data analyses

All statistical analyses and visualization was done in R version 4.3.2 (R core team, 2023) using the tidyverse suite of packages (Wickham *et al.*, 2019). Linear mixed modelling was performed using the lme4 package (Bates *et al.*, 2015) and F-statistics, degrees of freedom and p-values were calculated using the lmerTest package (Kuznetsova *et al.*, 2017). Fixed factors were standardized to a mean of 0 and a standard deviation of 0.5 using the standardize() command of the arm package (Gelman and Su, 2022). Fixed factors included water temperature (see below for details), body mass, OAC DO concentration, fish species and the interactions between these variables. Model selection was done in two stages utilizing AIC calculated by the MuMin package (Bartoń, 2022). In the first stage of model selection, OAC water temperature variables were compared: maximum, mean and variance in temperature calculated over 1–7 days prior to the trial date. The temperature variable that explained the most variation (with the lowest AIC score) was selected, and a second stage of model selection proceeded with the selected temperature variable and the other fixed effects listed above. All logical combinations of these models were compared; fixed factors were removed if their removal decreased the AIC by > 2. This process was done for each response variable (CT_max_, T_ag_, *P*_crit_, and LOE; see Supplementary Tables S2–9 for AIC results). A random factor of date was retained in all models to account for non-independence of measurements made on the same day. For CT_max_ and T_ag_, we included trial nested within date as a random factor, but trial explained very little variation in the model and was dropped to improve model fit. The total amount of variation explained by the models is presented as conditional *R*^2^, while the amount of variation explained by fixed factors is presented as marginal *R*^2^ (calculated by the r2_nakagawa() command of the performance package; Lüdecke et al., 2021).

## Results

### Overview

From May to September 2021, 81 Pugnose Shiner and 131 Blackchin Shiner were measured for CT_max_ (Table 1), and 24 Pugnose Shiner and 48 Blackchin Shiner were measured for respirometry (*P*_crit_ and LOE; Table 2). The difference in sample sizes between species reflects the difficulty in fishing for the Threatened Pugnose Shiner. Shoals of blackline shiners were targeted during sampling and the proportion of Pugnose Shiner in the shoals was much smaller than that of Blackchin Shiner, but we found that the likelihood of capturing Pugnose Shiner was highest when we targeted shoals of blackline shiners.

**Table 2:** Biometric data (mean ± s.e.) for Blackchin Shiner and Pugnose Shiner used in hypoxia tolerance measurements

**Species**	**Month**	**N**	**Mass (g)**	**SL (cm)**	**CF (g/cm** ^**3**^ **)**	***P***_**crit**_ **(% a.s.)**	**LOE (% a.s.)**
Blackchin Shiner	Jun	6	1.75 ± 0.21	4.5 ± 0.2	1.92 ± 0.09	37.7 ± 2.47	16.20 ± 3.35
	Jul	9	1.28 ± 0.07	4.2 ± 0.1	1.67 ± 0.04	25.3 ± 4.29	12.30 ± 1.55
	Aug	25	1.36 ± 0.05	4.4 ± 0.0	1.54 ± 0.02	23.1 ± 1.86	9.58 ± 0.45
	Sept	8	1.40 ± 0.08	4.6 ± 0.1	1.48 ± 0.03	18.9 ± 2.23	7.33 ± 0.54
Pugnose Shiner	Jun	7	1.36 ± 0.14	4.2 ± 0.1	1.80 ± 0.04	32.3 ± 3.36	14.6 ± 1.64
	Jul	7	0.98 ± 0.07	3.8 ± 0.2	1.87 ± 0.18	31.2 ± 4.02	17.10 ± 1.77
	Aug	6	1.19 ± 0.06	4.2 ± 0.0	1.59 ± 0.06	28.8 ± 4.77	12.3 ± 1.17
	Sept	4	1.11 ± 0.04	3.2 ± 0.0	1.50 ± 0.04	23.9 ± 5.90	9.67 ± 1.93

Fulton’s condition factor (CF) was calculated as Mass divided by Standard Length (SL) cubed.

All of the fish used in this study were all assumed to be adults based on their size. Both species spawn during the early summer, and it is possible they were reproductively active during measurements. This could have led to the fish allocating much of their energy to reproduction causing an increase in MO_2_ and therefore *P*_crit_. Both species reproduce during the same season; however, so it is unlikely that the reproductive activity has affected the comparative framework of this study.

### Environmental variables

**Figure 2:**
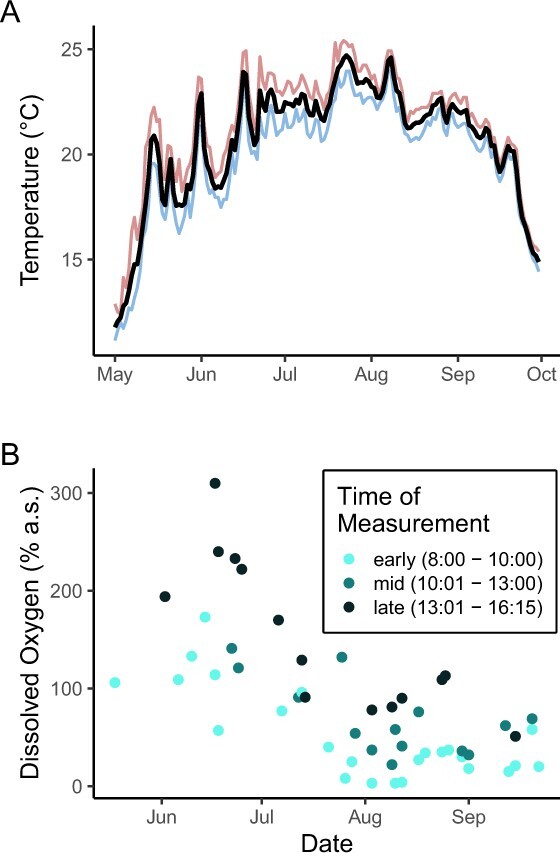
Water temperature (A) and dissolved oxygen concentration (DO, B) of the Old Ausable Channel during the study period. Surface water temperature data were collected at 15-minute intervals by a logger placed near the field site (see Methods for details). Data are presented as mean (black line), min (blue line) and max (red line) per day. DO measurements were made manually beside the research trailer (see methods). Each dot represents an individual measurement and is colour coded by time of day.

During this study, the surface temperature in the OAC varied from a minimum of 11.1°C at the beginning of May and peaked at a maximum of 25.4°C in late July (Fig. 2A). DO in the channel tended to decrease over the course of the field season and cycled daily such that DO was lower in the early morning and was higher later in the day (Fig. 2B).

### Thermal tolerance

**Figure 3:**
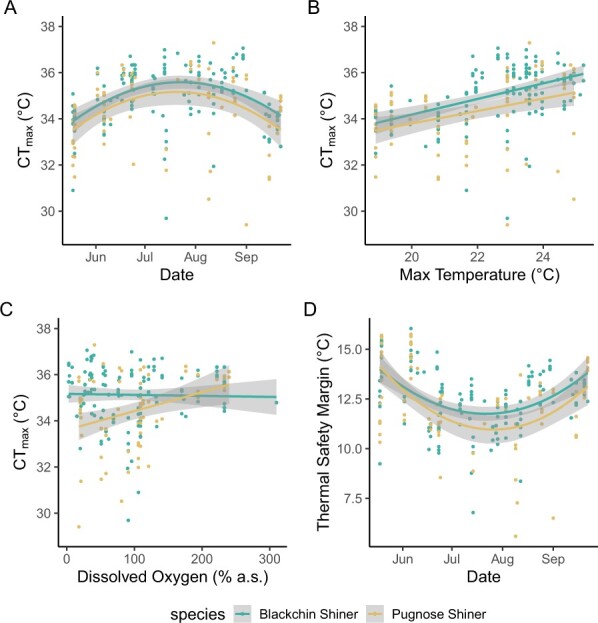
Critical thermal maximum (CT_max_) measured in Blackchin Shiner (blue) and Pugnose Shiner (yellow) over the course of the study (A). Panel B shows the effect of temperature (maximum surface temperature in the channel of the 1 day before CT_max_ measurement), while (C) shows the differential effect of dissolved oxygen (DO) between the species, where Pugnose Shiner is negatively affected by lower DO, while no effect is seen on Blackchin Shiner. Panel D presents thermal safety margin over the course of the field season. Coloured lines represent the line of best fit (second degree polynomial for (A, D, and linear regression for B, C) with the shaded area representing the 95% CI.

The final linear mixed model selected for CT_max_ (see methods for details) included mass, species, DO and maximum temperature of the 1-day period preceding the trial as fixed effects. It should be noted that maximum temperature and the average temperature of the 1-day period preceding the trial explained a similar amount of variation in CT_max_, but only one was included in the model because they were highly correlated. The effect of species and temperature were statistically significant (*P* < 0.05; Table 3), while a weaker, nonsignificant effect of DO and an interaction between DO and species were detected. Overall, Pugnose Shiner had a lower CT_max_ than Blackchin Shiner, and both species were affected similarly by temperature, with a positive relationship with maximum temperature and CT_max_ (Table 3; Fig. 3B). For Pugnose Shiner, there was a positive relationship between DO and CT_max_ leading to a decline in CT_max_ under hypoxia, while Blackchin Shiner CT_max_ was not affected by DO (Table 3; Fig. 3C). Thermal safety margin varied over the course of the field season, but generally remaining above 10°C (Fig. 3D).

**Table 3:** Results from linear mixed modelling of critical thermal maximum (CT_max_), critical oxygen tension (*P*_crit_) and loss of equilibrium (LOE) data for Blackchin Shiner and Pugnose Shiner over the course of the study

Response variable	*R* ^2^_marginal_	*R* ^2^_conditional_	Fixed factor	Model estimate	DF	*F*_statistic_	*P*-value
*CT_max_ ~ Mass + Species + DO + maxTemp_1day + Species:DO + (1|Date)*							
CT_max_	0.181	0.422	Intercept	34.81			
			Mass	−0.369	1, 194	3.323	0.070
			**Species**	**−0.542**	**1, 189**	**7.278**	**0.008**
			DO	0.038	1, 91	0.023	0.880
			**maxTemp_1day**	**1.143**	**1, 34**	**9.905**	**0.003**
			Species:DO	0.603	1, 189	2.808	0.095
*T_ag_ ~ Mass + Species + DO + maxTemp_5day + Species:DO + (1|Date)*							
T_ag_	0.199	0.346	Intercept	27.636			
			**Mass**	**−1.010**	**1, 182**	**4.323**	**0.039**
			**Species**	**−0.964**	**1, 183**	**4.236**	**0.041**
			DO	0.720	1, 62	1.786	0.186
			**maxTemp_1day**	**2.278**	**1, 46**	**15.554**	**<0.001**
			**Species:DO**	**2.083**	**1, 182**	**6.016**	**0.015**
*Pcrit ~ Mass + Species + DO + varTemp_5day + Mass:Species + (1|Date)*							
P_crit_	0.299	0.432	Intercept	1.398			
			**Mass**	**0.098**	**1, 72**	**5.414**	**0.023**
			Species	0.085	1, 51	2.838	0.098
			DO	−0.099	1, 21	1.817	0.192
			**varTemp_5day**	**0.211**	**1, 16**	**9.868**	**0.006**
			Mass:Species	0.143	1, 70	2.168	0.145
*LOE ~ Species + DO + varTemp_7day + Species:DO + (1|Date)*							
LOE	0.499	0.549	Intercept	11.837			
			**Species**	**1.859**	**1, 39**	**8.830**	**0.033**
			DO	0.708	1, 16	0.008	0.612
			**varTemp_7day**	**5.033**	**1, 14**	**16.143**	**0.001**
			**Species:DO**	**−3.585**	**1, 21**	**4.516**	**0.046**

Models were selected based on AIC (see methods for details), and fixed factors were standardized to a mean of 0 and s.d. of 0.5 prior to model fitting. Interactions between fixed factors are represented with a colon. Bold type indicates significance at *α* = 0.05. Degrees of freedom and *F*-statistics were calculated using type III ANOVA with Satterthwaite’s method.

**Figure 4:**
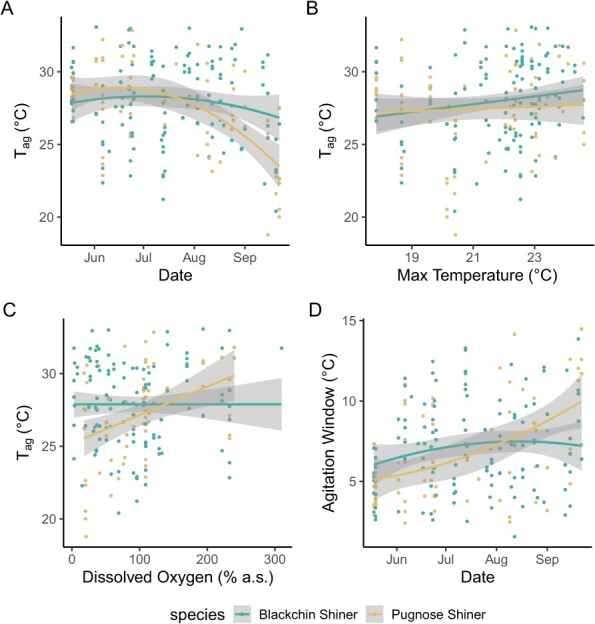
Agitation temperature (*T*_ag_) measured in Blackchin Shiner (blue) and Pugnose Shiner (yellow) over the course of the study (A). Panel B shows the effect of temperature (maximum surface temperature in the channel of the 5 days before *T*_ag_ measurement), while (C) shows the differential effect of dissolved oxygen (DO) between the species, where Pugnose Shiner is negatively affected by lower DO, while no effect is seen on Blackchin Shiner. (D) presents agitation window (difference between critical thermal maximum [CT_max_] and T_ag_) over the course of the field season. Coloured lines represent the line of best fit (second degree polynomial for (A) and (D), and linear regression for B, C) with the shaded area representing the 95% CI.

Of the 212 fish measured for CT_max_, eight Pugnose Shiner and three Blackchin Shiner were agitated throughout the entire trial and excluded from analysis of *T*_ag_. The final linear mixed model selected for *T*_ag_ contained the same fixed factors as that of CT_max_ except that the maximum temperature across the 5 days preceding measurement was selected as the best temperature predictor. Body mass, species, temperature and the interaction between species and DO were all significant predictors of *T*_ag_ (Table 3). *T*_ag_ had a positive relationship with channel temperature (4B): larger fish agitated at a lower temperature than smaller fish; and Pugnose Shiner agitated at a lower temperature than Blackchin Shiner (Fig. 4A). For Pugnose Shiner, there was also a positive relationship between *T*_ag_ and DO, such that fish agitated earlier under hypoxia, while Blackchin Shiner *T*_ag_ was unaffected by DO (Fig. 4C). Agitation window (difference between CT_max_ and *T*_ag_) was variable, measuring between 1.57 and 14.48 (Fig. 4D).

### Hypoxia tolerance

**Figure 5:**
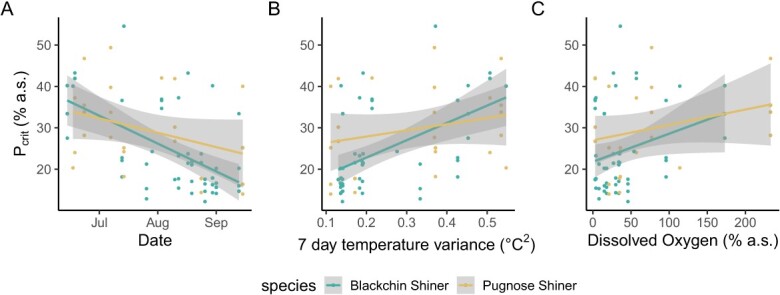
Critical oxygen tension (*P*_crit_) measured in Blackchin Shiner (blue) and Pugnose Shiner (yellow) over the course of the study (A). (B) shows the effect of temperature (variance in surface temperature in the channel of the 5 days before *P*_crit_ measurement), while (C) shows the weak effect of dissolved oxygen (DO), which was not statistically significant (Table 3). Coloured lines represent the line of best fit (linear regression) with the shaded area representing the 95% CI.

The final linear model selected for *P*_crit_ included body mass, species, DO, an interaction between mass and species, and the variance in temperature in the 5-day period before *P*_crit_ measurement. Only body mass and temperature variance (squared deviation from the mean) had a significant effect on *P*_crit_ such that larger fish had a higher *P*_crit_, and more variable temperatures led to higher *P*_crit_ (Table 3; Fig. 5B).

**Figure 6:**
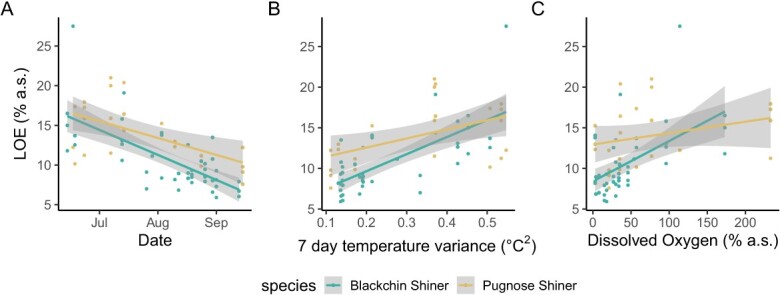
Loss of equilibrium during hypoxia (LOE) measured in Blackchin Shiner (blue) and Pugnose Shiner (yellow) over the course of the study (A). (B) shows the effect of temperature (variance in surface temperature in the channel of the 7 days before LOE measurement), while (C) shows the differential effect of dissolved oxygen (DO) between the species, where Blackchin Shiner is more affected by DO, than Pugnose Shiner (Table 3). Coloured lines represent the line of best fit (linear regression) with the shaded area representing the 95% CI.

The final linear model selected for LOE included body mass, species, DO, an interaction between species and DO and the variance in temperature in the 7-day period before LOE measurement. Species, the interaction between DO and species, and temperature variance all significantly affected LOE (Table 3). Overall, Pugnose Shiner lost equilibrium at higher levels of DO than Blackchin Shiner and were not affected by DO levels in the channel; whereas, Blackchin Shiner showed more plasticity in response to DO levels where LOE was lower when DO levels in the channel were more hypoxic (Fig. 6C).

Hypoxia tolerance metrics (*P*_crit_ and LOE) were positively correlated among individuals of both species (Pearson’s *R* = 0.719 ± 0.181 95% CI, p < 0.001 for Blackchin Shiner and Pearson’s *R* = 0.592 ± 0.354, p < 0.01 for Pugnose Shiner; Supplementary Fig. S2).

### Correlations between tolerance traits

Although measurements of thermal and hypoxia tolerance traits were made on different fish, both traits were usually measured on the same day and correlations between traits were made by averaging responses per day. Due to the lower sample size of Pugnose Shiner, this was only done for the Blackchin Shiner data. We found a negative correlation between CT_max_ and *P*_crit_ (Pearson’s *r* = −0.581, *P* = 0.015; Supplementary Fig. S3), but no correlation between CT_max_ and LOE (Pearson’s *r* = −0.291, *P* = 0.220; Supplementary Fig. S3). However, it should be noted that the relationship between CT_max_ and *P*_crit_ is primarily driven by a single datapoint that represents data for a single individual for respirometry and four individuals for CT_max_. When this one data point is removed, the correlation is not maintained (Pearson’s *r* = −0.147, *P* = 0.587).

## Discussion

As the planet warms due to climate change, and summer heat waves become more severe in Canada, understanding how fish respond to warming and other associated environmental stressors in the wild is imperative for informing their conservation. Use of surrogate species has been suggested to effectively use conservation dollars; however, this has not been widely validated. The present study aimed to address both the vulnerability of the Threatened Pugnose Shiner to two environmental stressors (heat and hypoxia) as well as validate the use of the non-imperilled Blackchin Shiner as a surrogate for Pugnose Shiner.

To our knowledge, this is the first experiment to measure field physiological tolerances in both Pugnose Shiner and Blackchin Shiner, although prior studies of Pugnose Shiner have investigated tolerance to elevated temperature, hypoxia and turbidity in laboratory settings (Gray *et al.*, 2014, 2016; McDonnell *et al.*, 2021; Potts *et al.*, 2021b). Overall, this experiment found species-specific responses in tolerance to high temperature and hypoxia. We also found that plasticity in response to temperature change was similar between the species for all tolerance metrics, but the Threatened Pugnose Shiner had lower tolerance to both environmental stressors than the non-imperilled Blackchin Shiner. Moreover, the species differed in their responses to environmental DO. CT_max_ of Pugnose Shiner had a positive relationship with DO such that CT_max_ was lowered when the OAC was hypoxic, whereas there was no effect of DO on CT_max_ of Blackchin Shiner. The same pattern was also seen with behavioural thermal tolerance metric (*T*_ag_). Blackchin Shiner also showed plasticity of hypoxia tolerance (LOE) in response to changes in environmental DO, while Pugnose Shiner showed little plasticity. However, the less severe hypoxia tolerance metric, *P*_crit,_ did not differ between species. We discuss these results in a broader context below and conclude that caution should be taken when using Blackchin Shiner as a surrogate for Pugnose Shiner.

### Thermal tolerance

In this study, we found that CT_max_ in Pugnose Shiner was lower than Blackchin Shiner, but that the effect of temperature on CT_max_ was similar between species, i.e. both species showed evidence of seasonal acclimatization. McDonnell *et al*. (2021) found similar results through a review of CT_max_ values among fish species in the genus *Notropis* (a subset of which are now in the genus *Miniellus*), where Pugnose Shiner showed similar plasticity in CT_max_, but lower values overall when compared to other congeners. Our results confirm this trend of similar plasticity but overall lower thermal tolerance of Pugnose Shiner.

Previous work has also investigated the thermal tolerance of Pugnose Shiner in the laboratory setting after acclimation to multiple temperatures and DO levels (McDonnell *et al.*, 2021; Potts *et al.*, 2021b), while, to our knowledge, no previous studies have measured CT_max_ of Pugnose Shiner or Blackchin Shiner on fish tested directly after capture in the field. In the present study, we found CT_max_ values comparable to those previously measured in the lab. Pugnose Shiner CT_max_ ranged from 29.41 to 37.29°C, while environmental temperature maxima (maximum temperature for a given day) ranged from 18.90 to 25.22°C. Previous measures of CT_max_ on juvenile Pugnose shiner averaged 32.0 to 36.0°C for fish acclimated to 19 and 25°C (Potts *et al.*, 2021b), while adults had CT_max_ values of 33.0 to 34.8°C for fish acclimated to 20 and 25°C (McDonnell *et al.*, 2021).

Interestingly, thermal tolerance in Pugnose Shiner was affected by DO whereby *T*_ag_ was reached at lower temperatures and CT_max_ was lower when the channel was hypoxic. This was not the case for the Blackchin Shiner, which showed no apparent relationship between CT_max_ and DO or *T*_ag_ and DO. This suggests that when the environment becomes hypoxic, Pugnose Shiner’s normal behaviour is disrupted at lower temperatures, and the species is less tolerant to warming. The pattern for Pugnose Shiner also provides evidence for oxygen dependency of thermal tolerance in this species. The theory of oxygen and capacity-limited thermal tolerance (OCLTT) has been used to explain the link between DO and thermal tolerance wherein CT_max_ occurs due to aerobic energy demands exceeding its capacity to supply oxygen to tissues as temperatures increase (Pörtner, 2010). However, the universality of the OCLTT framework has been questioned, as the oxygen dependence of CT_max_ appears to be species specific (Ern *et al.*, 2016). Our results support this as CT_max_ appears to be oxygen dependent in Pugnose Shiner but not Blackchin Shiner. We therefore may expect Pugnose Shiner to follow the framework of the OCLTT, but not Blackchin Shiner.

While physiological tolerance to warming is important for performance and survival, behavioural responses to environmental change allow organisms to mitigate the effects of environmental stressors on a shorter timescale and as an immediate response to an environmental stressor. In this study, behaviour (*T*_ag_) was also assessed during CT_max_ trials and showed similar patterns to those found for CT_max_; Pugnose Shiner became agitated at lower temperatures than Blackchin Shiner and agitation was positively associated with DO only in Pugnose Shiner. *T*_ag_ represents a disruption to normal behaviour; accordingly, reaching *T*_ag_ at a higher temperature allows a fish to persist normally for longer as temperatures rise. Previous studies of Pugnose Shiner in the lab have shown similar patterns to those found here, with *T*_ag_ increasing in response to temperature acclimation and decreasing in response to acute hypoxia exposure (McDonnell *et al.*, 2021; Potts *et al.*, 2021b; Fortin-Hamel and Chapman, 2024).

### Hypoxia tolerance

In this experiment, we measured two indices of hypoxia tolerance, LOE and *P*_crit_, and found differing results for each. The DO level at which Pugnose Shiner lost equilibrium was higher than for Blackchin Shiner. In addition, Blackchin Shiner showed a higher level of seasonal acclimatization (plasticity) in LOE than Pugnose Shiner. *P*_crit_ was similar between species and positively related to mass and the variance environmental temperature (i.e. larger Blackchin Shiner showed less tolerance to hypoxia as estimated by *P*_crit_ and more variable environmental temperatures were associated with lower hypoxia tolerance). Together, these results indicate that Pugnose Shiner is less tolerant of extreme hypoxia than Blackchin Shiner as estimated by LOE, but that the two species did not differ in tolerance at higher levels of DO (near *P*_crit_).

*P*_crit_ is a less severe metric of hypoxia tolerance and represents the transition point where fish can no longer satisfy their aerobic energy requirements and begin to rely upon anaerobic metabolism with decreasing levels of DO, while LOE is an endpoint wherein death is imminent at levels below it. Because *P*_crit_ is a less severe endpoint, it was generally more variable than LOE and could have been influenced more by interindividual variation in activity during respirometry. Due to logistical constraints, respirometry trials were only conducted during daytime hours (~09:00–18:00). This precluded the measurement of standard metabolic rate overnight, and instead RMR was measured and used to calculate *P*_crit_, which also may have led to the higher variability of *P*_crit_ data (Reemeyer and Rees, 2019).

Gray *et al.* (2016) measured Pugnose Shiner RMR and *P*_crit_ after acclimation to clear and turbid (~7 NTU) conditions and measured a mean *P*_crit_ of 22% a.s. for clear-acclimated fish and 26.5% a.s. for turbid-acclimated fish held at 18°C. In the present study, *P*_crit_ for Pugnose Shiner was considerably higher and varied from 13.99 to 49.39% a.s. (mean of 29.68% a.s.) at 18.37 to 22.20°C (mean of 21.19°C). Few studies have directly compared field- and lab-measured *P*_crit_, but our data would suggest that *P*_crit_ values in the laboratory may overestimate tolerance to hypoxia. This may be due to factors not controlled for in a field setting, such as parasite load, disease, reproductive status, stress and diet (Turko *et al.*, 2023). Transport to, and maintenance in, the laboratory represents a bottleneck, with mortality often occurring. It is possible that the fish measured in this study, where fish were taken directly from the field to the research set up, encompass a broader and more representative range of fish phenotypes. Differences may also be due to temperature fluctuations, as the laboratory experiment used a constant 18°C, while the fish in the present study experienced fluctuating temperatures. It is also possible that differences between the two studies may relate to the source population of Pugnose Shiner, which differed between our study and Gray *et al.* (2016), or methodological variation as the average time to *P*_crit_ in the Gray *et al.* study was 8.7 h versus 1.2 h in the present study, and Gray *et al.* also used broken stick regression to calculate *P*_crit_ while we used a linear regression of MO_2_ values below RMR. It should be noted that these methodological variations may also have led to higher *P*_crit_ values being estimated in the Gray *et al.* data (broken stick regression has been shown to overestimate *P*_crit_; Reemeyer and Rees 2019), which would mean the true difference between the previous laboratory estimates and the ones measured here is actually greater.

We observed a decrease in DO in the OAC through the course of our study, with normoxic conditions in the spring that became hypoxic in the summer. Comparing our *P*_crit_ and LOE measures to the DO in the channel, we can see that environmental DO is often above LOE but drops below *P*_crit_ frequently in the summer months for both Pugnose Shiner and Blackchin Shiner. During this study, when DO in the channel dropped below 10% a.s., fish were observed performing aquatic surface respiration (J. Reemeyer, unpublished observation), wherein fish rise to the water’s surface and ventilate their gills with the top layer of water where oxygen has diffused from the air (Chapman and McKenzie, 2009). This suggests that fishes in the OAC are experiencing physiologically stressful DO conditions during the warmer months, as predicted by previous modelling analyses (Ziegler *et al.*, 2021).

We recognize that the sample size of Pugnose Shiner measured for respirometry is low. This is due to the rarity of Pugnose Shiner in the environment and the difficulty capturing them. However, great effort was made to spread the sampling of both species across the field season (and therefore across many temperatures and DO conditions). This should have led to an accurate comparison between species when patterns were compared across the field season.

### Blackchin shiner as a surrogate for Pugnose shiner

Surrogate species have broadly been suggested and implemented as a method of effectively targeting conservation dollars. More specifically, surrogate species have been put forth as a method of learning about an imperilled species’ environmental tolerance when access to that species is limited. To do this, a closely related species is selected, and it is assumed that the response to an environmental stressor may reflect that of the imperilled species (Wenger, 2008). We hypothesized that Blackchin Shiner would be a good surrogate species for Pugnose Shiner because of the evolutionary and ecological similarity between the species and the high likelihood of capturing them together in the field (Carlson, 1997; Haynes *et al.*, 2019; Potts *et al.*, 2021a). In this study, we set out to investigate the validity of this hypothesis by measuring individuals in the field experiencing the same environment (often captured together in the same seine haul). Overall, we find mixed results for the utilization of Blackchin Shiner as a surrogate for Pugnose Shiner. Responses to temperature were similar between species, however Pugnose Shiner tended to have a lower CT_max_ than Blackchin Shiner. However, the differential effect of DO on these species and the differing plasticity in hypoxia tolerance complicate the story and lead us to caution the use of Blackchin Shiner as a surrogate for Pugnose Shiner. Moreover, previous work comparing Pugnose Shiner to other blackline shiners found that imperilled and non-imperilled species differed in their behavioural and physiological responses to turbidity (Gray *et al.*, 2014, 2016). This further suggests that responses to environmental stressors tend to be species-specific and highlights the importance of studies exploring environmental thresholds for imperilled taxa.

### Broader conservation implications

In the present study, we found that Pugnose Shiner generally has a lower tolerance to elevated temperature and to extreme hypoxia than its non-imperilled congener Blackchin Shiner. This higher sensitivity of Pugnose Shiner may mean that it is more vulnerable to the predicted effects of climate change. In this species’ range, heat waves are predicted to increase in frequency, intensity and length (Meehl and Tebaldi, 2004), while DO levels are decreasing in freshwater systems due to human pressure (Jenny *et al.*, 2016). This is particularly concerning for a species with a small body size and relatively isolated populations, which may limit the ability of Pugnose Shiner to disperse into cooler or better oxygenated systems. The increased sensitivity of Pugnose Shiner also indicates that elevated temperature and hypoxia may already be drivers of imperilment in this species, as the more tolerant Blackchin Shiner is not thought to be experiencing population declines.

Our data also suggest that Pugnose Shiner is less tolerant to hypoxia than indicated by previous laboratory estimates (Gray *et al.*, 2016), as our measures of *P*_crit_ were, on average, higher than those measured by Gray *et al*. The degree that laboratory assays represent the physiological tolerance of fishes in the field is of great interest and has implications for the accuracy of predictions and conservation recommendations. Most physiological tolerance data are collected in a laboratory setting, as performing these assays in a field setting is challenging logistically and does not allow for controlling of environmental factors (Turko *et al.*, 2023). The difference in hypoxia tolerance seen here highlights the value of field measurements and possible issues with using laboratory measures to inform conservation of wild organisms.

The results of this study demonstrate that even very similar species living in the same environment can have differential responses to environmental stressors. While we recognize the use of surrogate species may be unavoidable in certain cases, e.g. where access to an imperilled species is limited, we conclude that the use of surrogate species should be validated on a case-by-case basis where possible, and that assumptions of representative responses should be confirmed before implementation in conservation actions. It is preferable to directly measure the target species where possible to gain more accurate insights into the environmental tolerance of a given species.

## Supplementary Material

Web_Material_coae071

## Data Availability

Data associated with this study are publicly available on Figshare (10.6084/m9.figshare.26035486).
