# Supplementary material for: Seasonal variation in thermal tolerance and hypoxia tolerance of a threatened minnow and a non-imperilled congener: a cautionary tale for surrogate species in conservation
Source: Conserv Physiol. 2024 Oct 16;12(1):coae071. doi: 10.1093/conphys/coae071 (PMC11482009; doi:10.1093/conphys/coae071)
Supplement: Web_Material_coae071 [file web_material_coae071.pdf]

Supplemental Information

Table S1. Checklist of 53 essential criteria for the reporting of methods for aquatic intermittent-flow respirometry. (Taken directly from Killen et al 2021)

Number	Criterion and Category	Response	Value (where required)	Units
	**EQUIPMENT, MATERIALS, AND SETUP**			
**1**	Body mass of animals at time of respirometry	Fish were weighed and measured before placement into the respirometer		
**2**	Volume of empty respirometers	58 ml		
**3**	How chamber mixing was achieved	A closed circulation loop with a water pump		
**4**	Ratio of net respirometer volume (plus any associated tubing in mixing circuit) to animal body mass	Min = 77.38, max = 24.11, mean = 42.81		
**5**	Material of tubing used in any mixing circuit	Tygon low gas permeability tubing		
**6**	Volume of tubing in any mixing circuit	The volume of the respirometer and tubing was measured together	N/A	
**7**	Confirm volume of tubing in any mixing circuit was included in calculations of oxygen uptake	Yes		
**8**	Material of respirometer (e.g. glass, acrylic, etc.)	Glass		
**9**	Type of oxygen probe and data recording	Witrox 4 (loligosystems.com) dissolved oxygen		
**10**	Sampling frequency of water dissolved oxygen	Once per second		
**11**	Describe placement of oxygen probe (in mixing circuit or directly in chamber)	Directly in chamber		
**12**	Flow rate during flushing and recirculation, or confirm that chamber returned to normoxia during flushing	Dissolved oxygen returned to normoxia during flushing. Flow rate of approximately 2 cm s^{-1} during flushing and 1 cm s^{-1} during recirculation.		
**13**	Timing of flush/closed cycles	Between 120 and 420 s (depending on size of fish)		
**14**	Wait (delay) time excluded from closed measurement cycles	30 s		
**15**	Frequency and method of probe calibration (for both 0 and 100% calibrations)	0% calibration was done using a sodium sulfite solution and 100% calibration was done using aerated water. Calibration occurred approximately every 3 weeks.		

**16**	State whether software temperature compensation was used during recording of water oxygen concentration	yes		
	**MEASUREMENT CONDITIONS**			
**17**	Temperature during respirometry	Temperature varied between trials but was maintained constant within a trial. Min = 18.37°C, max = 23.38°C, mean: 21.68°C		
**18**	How temperature was controlled	A pump with a metal coil loop submerged in an ice bath recirculated water from the system when the temperature exceeded a set point		
**19**	Photoperiod during respirometry	Fish were measured in the field (not held in the lab). Natural photoperiod during the time period of the study was ~12:12 light:dark.	N/A	
**20**	If (and how) ambient water bath was cleaned and aerated during measurement of oxygen uptake (e.g. filtration, periodic or continuous water changes)	Water was aerated using an airstone and air bubbler. Water was taken directly from the channel where fish were caught and not cleaned during the trial.		
**21**	Total volume of ambient water bath and any associated reservoirs	Approximately 24 L		
**22**	Minimum water oxygen dissolved oxygen reached during closed phases	The measurement period length was set to keep the minimum DO in the chambers above 80%		
**23**	State whether chambers were visually shielded from external disturbance	The water bath containing the respirometry chambers was covered in opaque plastic to shield the fish from exernal disturbance		
**24**	How many animals were measured during a given respirometry trial (i.e. how many animals were in the same water bath)	4 individuals per trial	4	individuals
**25**	If multiple animals were measured simultaneously, state whether they were able to see each other during measurements	Fish were able to see one another		
**26**	Duration of animal fasting before placement in respirometer	Fish were held overnight in a cooler before measurement	18 - 24	hours
**27**	Duration of all trials combined (number of days to measure all animals in the study)	4 months (June – September 2022)	4	months
**28**	Acclimation time to the laboratory (or time since capture for field studies) before respirometry measurements	Fish were measured 18 – 24 hours after capture	18 – 24	hours
	**BACKGROUND RESPIRATION**			
**29**	State whether background microbial respiration was measured and accounted for, and if so, method used (e.g. parallel measures with empty respirometry chamber, measurements before and after for all chambers while empty, both)	Background respiration was measured before and after every trial for all chambers.		
**30**	State if background respiration was measured at beginning and/or end, state how many slopes and for what duration	One slope was used for each before and after. The duration of the background measurement period was 1200 seconds.		
**31**	State how changes in background respiration were modelled over time (e.g. linear, exponential, parallel measures)	Background respiration was 0, so we did not need to account for it		

**32**	Level of background respiration (e.g. as a percentage of SMR)	0		
**33**	Method and frequency of system cleaning (e.g. system bleached between each trial, UV lamp)	System was cleaned with dilute bleach between every trial		
	**STANDARD OR ROUTINE METABOLIC RATE**			
**34**	Acclimation time after transfer to chamber, or alternatively, time to reach beginning of metabolic rate measurements after introduction to chamber	MO_2 measurements began immediately		
**35**	Time period, within a trial, over which oxygen uptake was measured (e.g. number of hours)	4-5 hours		
**36**	Value taken as SMR/RMR (e.g. quantile, mean of lowest 10 percent, mean of all values)	Mean of the lowest 10 percent		
**37**	Total number of slopes measured and used to derive metabolic rate (e.g. how much data were used to calculate quantiles)	Approximately 40 slopes (~4 used to calculate RMR)		
**38**	Whether any time periods were removed from calculations of SMR/RMR (e.g. data during acclimation, periods of high activity [e.g. daytime])	Only slopes measured before the prolonged closed period for P_{crit} measurement were used		
**39**	r^2 threshold for slopes used for SMR/RMR (or mean)	0.9 threshold		
**40**	Proportion of data removed due to being outliers below r-squared threshold	None		
	**MAXIMUM METABOLIC RATE**			
**41**	When MMR was measured in relation to SMR (i.e. before or after)	N/A		
**42**	Method used (e.g. critical swimming speed respirometry, swim to exhaustion in swim tunnel, or chase to exhaustion)	N/A		
**43**	Value taken as MMR (e.g. the highest rate of oxygen uptake value after transfer, average of highest values)	N/A		
**44**	If MMR measured post-exhaustion, length of activity challenge or chase (e.g. 2 min, until exhaustion, etc.)	N/A		
**45**	If MMR measured post-exhaustion, state whether further air-exposure was added after exercise	N/A		
**46**	If MMR measured post-exhaustion, time until transfer to chamber after exhaustion or time to start of oxygen uptake recording	N/A		
**47**	Duration of slopes used to calculate MMR (e.g. 1 min, 5 min, etc.)	N/A		
**48**	Slope estimation method for MMR (e.g. rolling regression, sequential discrete time frames)	N/A		

**49**	How absolute aerobic scope and/or factorial aerobic scope is calculated (i.e. using raw SMR and MMR, allometrically mass-adjusted SMR and MMR, or allometrically mass-adjusting aerobic scope itself)	N/A		
	**DATA HANDLING AND STATISTICS**			
**50**	Sample size	72 individuals	72	individuals
**51**	How oxygen uptake rates were calculated (software or script, equation, units, etc.)	Autoresp software		
**52**	Confirm that volume (mass) of animal was subtracted from respirometer volume when calculating oxygen uptake rates	Yes		
**53**	State whether analyses accounted for variation in body mass and describe any allometric mass-corrections or adjustments	The effect of body mass was explored by including it as a fixed factor in linear mixed modelling analyses		

Table S2: Overview of water temperature variable model selection for critical thermal maximum (CT_{max}) of Pugnose Shiner (*Miniellus anogenus*) and Blackchin Shiner *(Miniellus heterolepis*). Models are formatted in the syntax used in the lme4 package of R for linear mixed models. The (1|date) term refers to the random factor of date. Temperature variables include maximum (max), mean (avg), and variance (var) of water temperature (Temp) calculated over 1 to 7 days prior to CT_{max} measurement. AIC was calculated for each model and ΔAIC refers to the difference in AIC with the best fit model.

Model	AIC	ΔAIC
CT_{max} ~ maxTemp_1day + (1|date)	679.67	0.00
CT_{max} ~ avgTemp_1day + (1|date)	680.22	0.55
CT_{max} ~ maxTemp_6day + (1|date)	682.00	2.32
CT_{max} ~ maxTemp_7day + (1|date)	682.07	2.40
CT_{max} ~ avgTemp_2day + (1|date)	682.36	2.69
CT_{max} ~ maxTemp_5day + (1|date)	682.47	2.79
CT_{max} ~ maxTemp_2day + (1|date)	682.57	2.90
CT_{max} ~ maxTemp_4day + (1|date)	682.68	3.01
CT_{max} ~ maxTemp_3day + (1|date)	683.01	3.34
CT_{max} ~ avgTemp_3day + (1|date)	683.20	3.52
CT_{max} ~ avgTemp_4day + (1|date)	684.14	4.47
CT_{max} ~ avgTemp_5day + (1|date)	684.47	4.80
CT_{max} ~ avgTemp_6day + (1|date)	684.58	4.90
CT_{max} ~ avgTemp_7day + (1|date)	684.81	5.13
CT_{max} ~ (1|date)	688.91	9.24
CT_{max} ~ varTemp_1day + (1|date)	689.99	10.32
CT_{max} ~ varTemp_4day + (1|date)	690.40	10.73
CT_{max} ~ varTemp_3day + (1|date)	690.42	10.75
CT_{max} ~ varTemp_2day + (1|date)	690.75	11.08
CT_{max} ~ varTemp_5day + (1|date)	690.84	11.17
CT_{max} ~ varTemp_7day + (1|date)	690.91	11.24
CT_{max} ~ varTemp_6day + (1|date)	690.91	11.24
CT_{max} ~ 1	755.11	75.44

Table S3: Overview of second stage of model selection for critical thermal maximum (CT_{max}). Models include most possible combinations of the fixed factors of interest: mass, species, dissolved oxygen (DO), maximum temperature 1 day before measurement of CT_{max} (maxTemp_1day). The (1|date) term refers to the random factor of date. AIC was calculated for each model and ΔAIC refers to the difference in AIC with the best fit model.

Model	AIC	ΔAIC
CT_{max} ~ mass + species + DO + species:DO + maxTemp_1d + (1|date)	629.06	0.00
CT_{max} ~ mass + species + DO + maxTemp_1day + (1|date)	629.81	0.75
CT_{max} ~ mass + species + DO + mass:species + maxTemp_1day + (1|date)	631.77	2.70
CT_{max} ~ DO + maxTemp_1day + (1|date)	632.29	3.23
CT_{max} ~ mass + species + DO + species:DO + (1|date)	635.82	6.76
CT_{max} ~ mass + species + DO + (1|date)	636.95	7.88
CT_{max} ~ DO + (1|date)	638.88	9.82
CT_{max} ~ mass + species + DO + mass:species + (1|date)	638.94	9.88
CT_{max} ~ mass + species + maxTemp_1day + (1|date)	676.77	47.70
CT_{max} ~ species + maxTemp_1day + (1|date)	677.76	48.70
CT_{max} ~ maxTemp_1day + (1|date)	680.22	51.16
CT_{max} ~ mass + maxTemp_1day + (1|date)	681.93	52.86
CT_{max} ~ mass + species + (1|date)	686.18	57.12
CT_{max} ~ species + (1|date)	686.55	57.49
CT_{max} ~ mass + (1|date)	690.76	61.70

Table S4: Overview of temperature variable model selection for agitation temperature (T_{ag}). Models are formatted in the syntax used in the lme4 package of R for linear mixed models. The (1|date) term refers to the random factor of date. Temperature variables include maximum (max), mean (avg), and variance (var) of temperature (Temp) calculated over 1 to 7 days prior to T_{ag} measurement. AIC was calculated for each model and ΔAIC refers to the difference in AIC with the best fit model.

Model	AIC	ΔAIC
T_{ag} ~ maxTemp_5day + (1|date)	970.35	0.00
T_{ag} ~ maxTemp_6day + (1|date)	970.53	0.18
T_{ag} ~ maxTemp_7day + (1|date)	971.48	1.14
T_{ag} ~ maxTemp_4day + (1|date)	972.16	1.82
T_{ag} ~ maxTemp_3day + (1|date)	973.90	3.56
T_{ag} ~ maxTemp_2day + (1|date)	974.70	4.35
T_{ag} ~ avgTemp_3day + (1|date)	977.36	7.02
T_{ag} ~ avgTemp_4day + (1|date)	977.41	7.07
T_{ag} ~ avgTemp_5day + (1|date)	977.70	7.35
T_{ag} ~ maxTemp_1day + (1|date)	977.76	7.41
T_{ag} ~ varTemp_5day + (1|date)	977.76	7.41
T_{ag} ~ avgTemp_2day + (1|date)	977.94	7.59
T_{ag} ~ varTemp_4day + (1|date)	977.95	7.60
T_{ag} ~ varTemp_7day + (1|date)	977.97	7.63
T_{ag} ~ varTemp_6day + (1|date)	978.49	8.14
T_{ag} ~ avgTemp_6day + (1|date)	978.69	8.34
T_{ag} ~ avgTemp_1day + (1|date)	978.96	8.62
T_{ag} ~ varTemp_3day + (1|date)	978.99	8.64
T_{ag} ~ avgTemp_7day + (1|date)	978.99	8.64
T_{ag} ~ varTemp_2day + (1|date)	979.88	9.53
T_{ag} ~ (1|date)	981.01	10.67
T_{ag} ~ varTemp_1day + (1|date)	981.36	11.01
T_{ag} ~ 1	981.90	11.56

Table S5: Overview of second stage of model selection for critical thermal maximum (T_{ag}). Models include most possible combinations of the fixed factors of interest: mass, species, dissolved oxygen (DO), maximum temperature in the 5 days before measurement of T_{ag} (maxTemp_1day). The (1|date) term refers to the random factor of date. AIC was calculated for each model and ΔAIC refers to the difference in AIC with the best fit model.

Model	AIC	ΔAIC
T_{ag} ~ mass + species + DO + species:DO + maxTemp_5d + (1|date)	891.59	0.00
T_{ag} ~ mass + species + DO + mass:species + maxTemp_5day + (1|date)	892.42	0.83
T_{ag} ~ mass + species + DO + maxTemp_5day + (1|date)	895.51	3.92
T_{ag} ~ DO + maxTemp_5day + (1|date)	896.18	4.60
T_{ag} ~ mass + species + DO + species:DO + (1|date)	903.00	11.41
T_{ag} ~ mass + species + DO + mass:species + (1|date)	903.41	11.82
T_{ag} ~ DO + (1|date)	908.09	16.50
T_{ag} ~ mass + species + DO + (1|date) 12	908.35	16.76
T_{ag} ~ maxTemp_5day + (1|date)	970.35	78.76
T_{ag} ~ mass + species + maxTemp_5day + (1|date)	970.75	79.16
T_{ag} ~ mass + maxTemp_5day + (1|date)	970.99	79.40
T_{ag} ~ species + maxTemp_5day + (1|date)	971.70	80.11
T_{ag} ~ mass + (1|date)	982.59	91.00
T_{ag} ~ species + (1|date)	982.64	91.05
T_{ag} ~ mass + species + (1|date)	982.65	91.07

Table S6: Overview of temperature variable model selection for critical oxygen tension (P_{crit}). Models are formatted in the syntax used in the lme4 package of R for linear mixed models. The (1|date) term refers to the random factor of date. Temperature variables include maximum (max), mean (avg), and variance (var) of temperature (Temp) calculated over 1 to 7 days prior to P_{crit} measurement, as well as the temperature maintained during the duration of the trial (trialTemp). AIC was calculated for each model and ΔAIC refers to the difference in AIC with the best fit model.

**Model**	**AIC**	**ΔAIC**
P_{crit} ~ varTemp_5day + (1|date)	533.19	0.00
P_{crit} ~ varTemp_6day + (1|date)	533.94	0.75
P_{crit} ~ varTemp_7day + (1|date)	535.20	2.02
P_{crit} ~ varTemp_4day + (1|date)	535.76	2.57
P_{crit} ~ varTemp_3day + (1|date)	536.77	3.58
P_{crit} ~ varTemp_1day + (1|date)	537.05	3.86
P_{crit} ~ varTemp_2day + (1|date)	537.94	4.75
P_{crit} ~ (1|date)	542.27	9.08
P_{crit} ~ maxTemp_6d + (1|date)	542.52	9.34
P_{crit} ~ maxTemp_5d + (1|date)	542.70	9.51
P_{crit} ~ maxTemp_2d + (1|date)	542.73	9.54
P_{crit} ~ maxTemp_7d + (1|date)	542.85	9.66
P_{crit} ~ maxTemp_3d + (1|date)	542.93	9.74
P_{crit} ~ maxTemp_4d + (1|date)	543.03	9.84
P_{crit} ~ maxTemp_1d + (1|date)	543.04	9.86
P_{crit} ~ avgTemp_1d + (1|date)	543.96	10.77
P_{crit} ~ avgTemp_7d + (1|date)	544.09	10.90
P_{crit} ~ avgTemp_6d + (1|date)	544.12	10.93
P_{crit} ~ avgTemp_2d + (1|date)	544.13	10.94
P_{crit} ~ avgTemp_5d + (1|date)	544.13	10.94
P_{crit} ~ avgTemp_4d + (1|date)	544.21	11.03
P_{crit} ~ trialTemp + (1|date)	544.26	11.07
P_{crit} ~ avgTemp_3d + (1|date)	544.27	11.08
P_{crit} ~ 1	548.26	15.07

Table S7: Overview of second stage of model selection for critical oxygen tension (P_{crit}). Models include most possible combinations of the fixed factors of interest: mass, species, dissolved oxygen (DO), variance of temperature of the 5 days before measurement of P_{crit} (varTemp_5day). The (1|date) term refers to the random factor of date. AIC was calculated for each model and ΔAIC refers to the difference in AIC with the best fit model.

**Model**	**AIC**	**ΔAIC**
P_{crit} ~ mass + varTemp_5d + (1|date)	533.04	0.00
P_{crit} ~ varTemp_5d + (1|date)	533.19	0.15
P_{crit} ~ mass + species + mass:species + DO + varTemp_5d + (1|date)	534.04	1.00
P_{crit} ~ mass + species + varTemp_5d + (1|date)	534.14	1.09
P_{crit} ~ species + varTemp_5d + (1|date)	534.84	1.80
P_{crit} ~ DO + varTemp_5d + (1|date)	534.98	1.94
P_{crit} ~ mass + species + DO + varTemp_5d + (1|date)	535.11	2.07
P_{crit} ~ mass + species + DO + species:DO + varTemp_5d + (1|date)	536.84	3.80
P_{crit} ~ mass + (1|date)	539.52	6.48
P_{crit} ~ DO + (1|date)	539.87	6.83
P_{crit} ~ mass + species + DO + (1|date)	540.41	7.37
P_{crit} ~ mass + species + (1|date)	540.53	7.49
P_{crit} ~ mass + species + mass:species + DO + (1|date)	540.95	7.91
P_{crit} ~ mass + species + DO + species:DO + (1|date)	541.63	8.59
P_{crit} ~ species	544.17	11.13

Table S8: Overview of temperature variable model selection for loss of equilibrium (LOE). Models are formatted in the syntax used in the lme4 package of R for linear mixed models. The (1|date) term refers to the random factor of date. Temperature variables include maximum (max), mean (avg), and variance (var) of temperature (Temp) calculated over 1 to 7 days prior to LOE measurement, as well as the temperature maintained during the duration of the trial (trialTemp). AIC was calculated for each model and ΔAIC refers to the difference in AIC with the best fit model.

Model	AIC	ΔAIC
LOE ~ varTemp_7d + (1|date)	351.42	0.00
LOE ~ varTemp_6d + (1|date)	352.29	0.88
LOE ~ varTemp_5d + (1|date)	353.85	2.44
LOE ~ varTemp_4d + (1|date)	355.78	4.36
LOE ~ varTemp_3d + (1|date)	358.48	7.06
LOE ~ varTemp_2d + (1|date)	361.28	9.86
LOE ~ varTemp_1d + (1|date)	364.34	12.92
LOE ~ maxTemp_2d + (1|date)	367.83	16.42
LOE ~ maxTemp_1d + (1|date)	368.36	16.94
LOE ~ maxTemp_3d + (1|date)	368.60	17.18
LOE ~ (1|date)	368.66	17.25
LOE ~ maxTemp_4d + (1|date)	369.07	17.66
LOE ~ maxTemp_5d + (1|date)	369.13	17.72
LOE ~ maxTemp_6d + (1|date)	369.15	17.73
LOE ~ maxTemp_7d + (1|date)	369.37	17.95
LOE ~ avgTemp_1d + (1|date)	369.75	18.33
LOE ~ avgTemp_7d + (1|date)	369.90	18.48
LOE ~ trialTemp + (1|date)	369.92	18.50
LOE ~ avgTemp_6d + (1|date)	370.08	18.66
LOE ~ avgTemp_5d + (1|date)	370.17	18.75
LOE ~ avgTemp_4d + (1|date)	370.37	18.95
LOE ~ avgTemp_2d + (1|date)	370.47	19.06
LOE ~ avgTemp_3d + (1|date)	370.64	19.22
LOE ~ 1	386.49	35.08

Table S9: Overview of second stage of model selection for loss of equilibrium (LOE). Models include most possible combinations of the fixed factors of interest: mass, species, dissolved oxygen (DO), variance of temperature of the 7 days before measurement of LOE (varTemp_7day). The (1|date) term refers to the random factor of date. AIC was calculated for each model and ΔAIC refers to the difference in AIC with the best fit model.

Model	AIC	ΔAIC
LOE ~ species + varTemp_7d + (1|date)	350.32	0.00
LOE ~ mass + species + DO + species:DO + varTemp_7d + (1|date)	350.77	0.45
LOE ~ mass + species + varTemp_7d + (1|date)	350.99	0.67
LOE ~ mass + varTemp_7d + (1|date)	351.17	0.85
LOE ~ varTemp_7d + (1|date)	351.42	1.10
LOE ~ mass + species + mass:species + DO + varTemp_7d + (1|date)	352.65	2.33
LOE ~ mass + species + DO + varTemp_7d + (1|date)	352.99	2.67
LOE ~ DO + varTemp_7d + (1|date)	353.39	3.07
LOE ~ mass + species + DO + species:DO + (1|date)	359.74	9.42
LOE ~ DO + (1|date)	362.11	11.79
LOE ~ mass + species + mass:species + DO + (1|date)	362.77	12.45
LOE ~ mass + species + DO + (1|date)	364.10	13.78
LOE ~ species + (1|date)	369.42	19.10
LOE ~ mass + (1|date)	370.60	20.28
LOE ~ mass + species + (1|date)	371.38	21.06

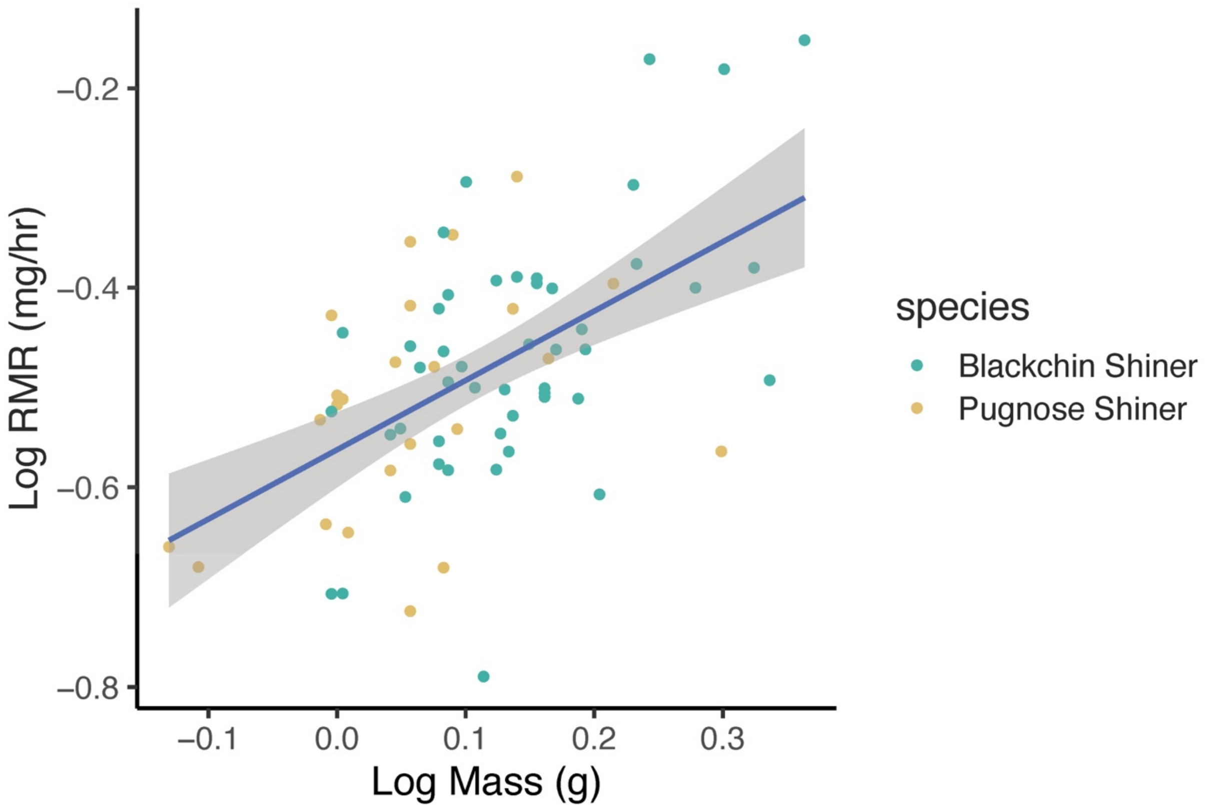

Figure S1: Relationship between $\log_{10}$ routine metabolic rate (RMR) and $\log_{10}$ body showing allometric scaling of RMR with body mass. Dots on the plot indicate individual with species coloured and singular trendline with equation. A linear regression of $\log_{10}$ RMR with mass and trial temperature as predictors was used to calculate the slope of the effect of body mass as 0.664 ± 0.246 (95% CI).

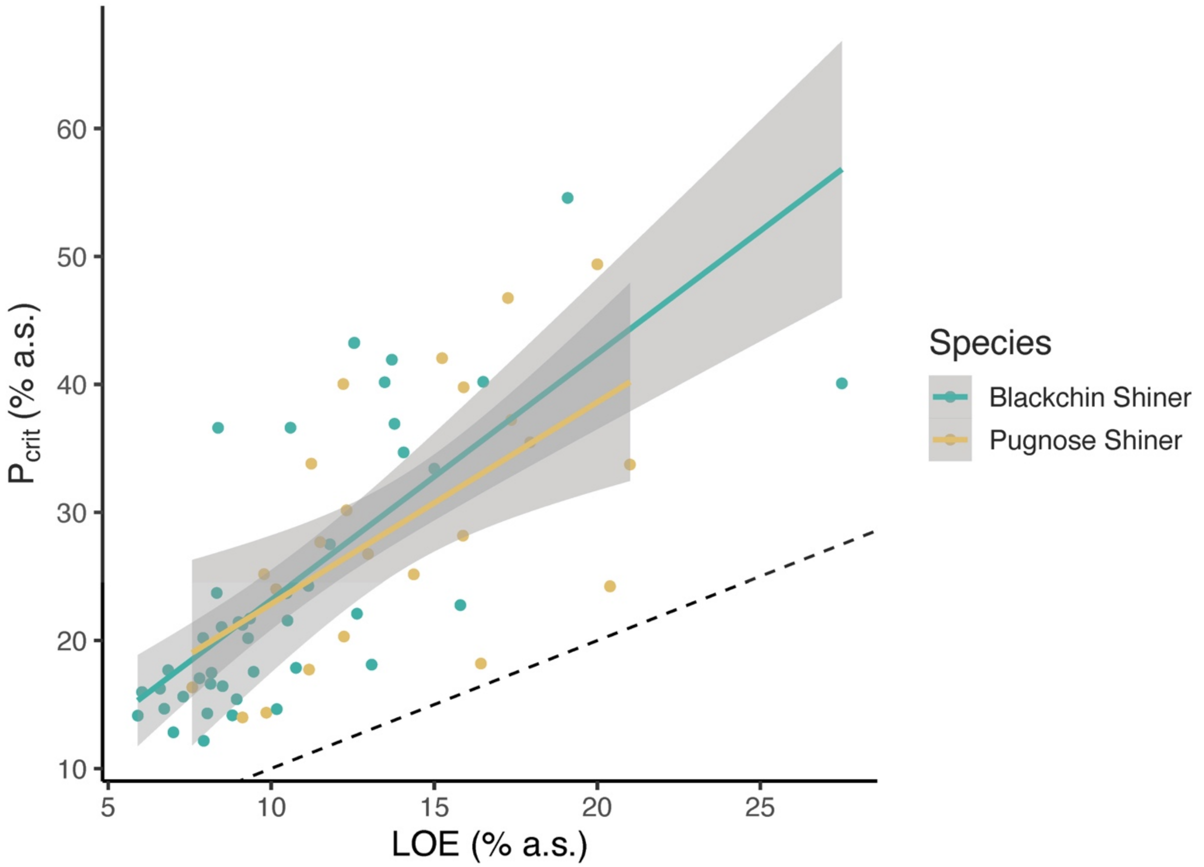

Figure S2: Relationship between hypoxia tolerance metrics (critical oxygen tension, P_{crit}; and loss of equilibrium, LOE). Data are coloured by species, with each dot representing an individual fish. Coloured lines show the linear regression of data for each species with the shaded gray area showing the 95% CI. The dashed line shows y=x. For Blackchin Shiner data the slope of the line was 1.922 ± 0.561 (95% CI) with a y intercept of 3.963 ± 6.359. For Pugnose Shiner data the slope of the line was 1.575 ± 0.916 with a y intercept of 7.111 ± 0.916. Pearson's correlation coefficient for Blackchin Shiner data was 0.719 ± 0.181 (95% CI; $p<0.001$), while the Pearson's R for Pugnose Shiner data was 0.592 ± 0.354 ($p<0.01$).

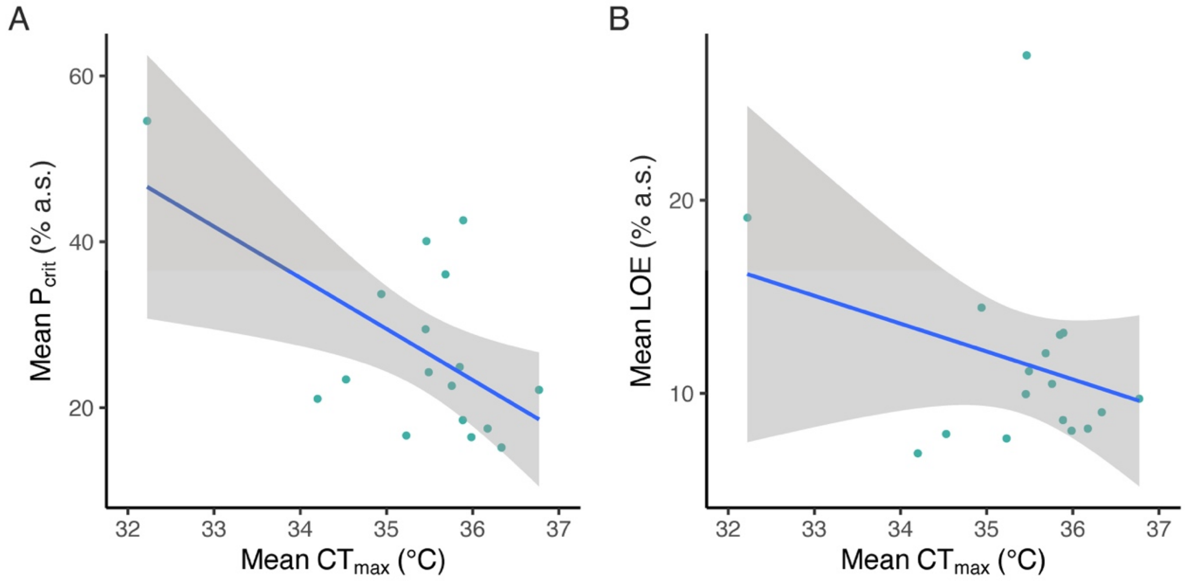

Figure S3: Relationship between thermal tolerance (critical thermal tolerance, CT_{max}) and hypoxia tolerance (critical oxygen tension, P_{crit}; and loss of equilibrium, LOE). Hypoxia tolerance data for Blackchin Shiner were binned and averaged by day, and the CT_{max} data corresponding to a given date was also averaged. The mean CT_{max} was then plotted against P_{crit} (panel A) and LOE (panel B). It should be noted that the overall negative slope of the line is driven by a single low data point in both panels. Pearson's correlation coefficient for CT_{max} against P_{crit} was -0.581 ± 0.249 (95% CI) and statistically significant (p=0.015), while the Pearson's r for CT_{max} and LOE was -0.291 ± 0.386 (95% CI) and nonsignificant (p=0.257).
